# Marrubiin

**DOI:** 10.3390/molecules18089049

**Published:** 2013-07-29

**Authors:** Olugbenga K. Popoola, Abdulrahman M. Elbagory, Farouk Ameer, Ahmed A. Hussein

**Affiliations:** Chemistry Department, University of Western Cape, Private Bag X17, Belleville 7535, South Africa; E-Mails: 3318925@uwc.ac.za (O.K.P.); 3376881@uwc.ac.za (A.M.E.); fameer@uwc.ac.za (F.A.)

**Keywords:** marrubiin, Lamiaceae, biological activity, lead compound, anti-inflammatory

## Abstract

The ethno-medicinal approach to drug discovery represents one of the most important sources of new and safe therapeutic agents to the challenges confronting modern medicine and daily life. Many of the traditionally important medicinal plants contain active molecules or ones that serve as precursors to biosynthesised secondary metabolites to which the biological activity could be attributed. Marrubiin is one such compound and is a potential valuable compound which exists in high concentrations in many traditionally important Lamiaceae species which have demonstrated excellent pharmacological properties with commendably high safety margins. Marrubiin’s attributes include a low turnover, high stability and little catabolism, which are core characteristics required for therapeutic compounds and nutraceuticals of economic importance. In addition, marrubiin is considered a potential substrate for potent active compounds *viz*; marrubiinic acid, and marrubenol. The contribution of marrubiin to drug discovery thus needs to be put into prospective due to its ready availability, high potential applications and ease of modification. In this short review we highlight the most important chemical and pharmacological aspects reported on marrubiin since it was discovered.

## 1. Introduction

Marrubiin is a widely known diterpenoid lactone that constitutes the bitter principle of the horehound and many other medicinal plants of the family Lamiaceae. It is one of the main constituents of *Marrubium vulgare*, *Leonotis leonurus* and *Leonotis nepetifolia* [1], which are used in several countries to treat different pathologies [2]. According to SciFinder Scholar, there are recently 130 published articles on marrubiin and these cover the chemical and biological aspects associated with the compound. It is a major constituent of many species of the genus *Marrubium* (Lamiaceae) and includes about 97 species found along the Mediterranean and temperate regions of the Eurasian zone. Subsequent research revealed that marrubiin is the most active diterpenoid responsible for the therapeutic properties observed from the species *Leonotis leonurus* [3,4], *Phlomis bracteosa* [5], *Marrubium deserti de Noe* [6], *Marrubium vulgare* [5,7,8,9,10,11,12,13,14,15,16,17,18,19], *Marrubium alysson* [20] and *Marrubium thessalum* [21,22]. Extensive pharmacological studies have demonstrated that marrubiin displays a suite of activities inclusing antinociceptive [12], antioxidant, antigenotoxic [3], cardioprotective [4], vasorelaxant [11], gastroprotective [7], antispasmodic [6], immunomodulating [23], antioedematogenic [9], analgesic [10,15], and antidiabetic properties [24].

## 2. Occurrence

Marrubiin was first isolated in Nature from *Marrubium vulgare* in 1842 and subsequently many other plants belonging to the same genus as well as some other members of family Lamiaceae [24] *viz*; *Leonotis leonurus* [4,5], *Phlomis bracteosa* [6], *Marrubium deserti de Noe* [3], *M. vulgare* [5,17,18,19], *M. velatinum* [25,26], *M. cylleneum* [25,26], *M. trachyticum* [27], *M. globosum* [28], *M. anisodon* [29], *M. sericeum* [20], *M. supinum* [20], *M. alysson* [20] and *M. thassalum* [21,22] have been reported to contain marrubiin. Only a single report mentioned the isolation of marrubiin from *Spiraea brahuica* belonging to the family Rosaceae [30].

## 3. Physical and Spectroscopic Data [31]

**Synonyms:** 6-[2-(3-Furanyl)ethyl]decahydro-6-hydroxy-2a,5a,7-trimethyl-2*H*-naphtho[1,8-*bc*]furan-2-one, 6-[2-(3-furanyl)ethyl]deca-hydro- 6-hydroxy-2a,5a,7-trimethyl-(2a*S*,5a*S*,6*R*,7*R*,8a*R*,8b*R*)-2*H*-naphtho[1,8-*bc*]furan-2-one, 15,16-epoxy-6β,9-dihydroxy-[2aS-(2aα,5aβ,6α,7α,8aα,8bα)]-8β*H*-labda-13(16),14-dien-19-oic acid γ-lactone (8CI); marrubiin (6CI); 15,16-epoxy-6β,9-dihydroxy-8β*H*-labda-13(16),14-dien-19-oic acid γ-lactone; NSC 36693. Physical properties and assignment of its ^1^HNMR signals are presented in Table 1.

## 4. Biosynthesis

Initially, the common mevalonic acid pathway was proposed for the biosynthesis of terpenoids including marrubiin [15,32], however, the results of various ^13^C-labelled glucose experiments by Rohmer were shown to be inconsistent with the expectations based on the mevalonate pathway. Consequently, the 1-deoxy-D-xylulose-5-phosphate/2-methyl-D-erythritol-4-phosphate (DOX/MEP) pathway has been proposed as the source of this compound in Nature [32,33,34]. The experimental data showed the production and accumulation of marrubiin in agreement with this newly discovered pathway [a non-mevalonate pathway] like in Eubacteria and Gymnospermae [35,36].

**Table 1:** Physical properties and ^1^H-NMR data of marrubiin [31].

Physical properties	Value	NMR data [37]
Molecular weight	332.43	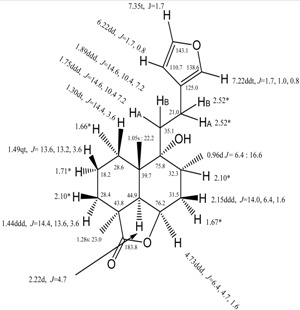 * Overlapped signals
Molecular formula	C_20_ H_28_ O_4_	
CAS Registry Number	465-92-9	
Molar volume	288 ± 3.0 cm^3^/mol(20 °C; 760 Torr)	
Density	1.152±0.06 g/cm^3^(20 °C; 760 Torr)	
Melting Point	~155–160 °C	
Freely Rotatable Bonds	4	
H Acceptors	4	
H Donors	1	
H Donor/ Acceptor sum	5	
LogP	3.796±0.414 (at 25 °C)	
[α]_D_:	+45.68° (acetone, 24 °C), +35.8 (CHCl_3_, 24 °C)	

Pre-furanic (e.g., premarrubiin) and furanic (e.g., marrubiin) labdanoids are widespread in the family Lamiaceae [38,39,40]. There are some ambiguities about the natural origin of the furanic labdanoids which possess a C-9 hydroxyl group and a furan ring in the side chain (like marrubiin) which are considered by some authors to be the final products in the biosynthetic pathway, while some others suggest that they are artefacts which arise from their corresponding prefuran labdanoids during or after the extraction or isolation process, by cleavage of the 9,13-epoxide bridge [41,42,43].

According to the recent literature [38], the detection of marrubiin in fresh plant material has been reported, together with the isolation of pre-furanic mixed with non-related furanic labdanoids [44,45,46,47], and these data indicate the existence of marrubiin as a natural compound inside the living organism and as an end product of a biosynthetic pathway. The transformation of premarrubiin into marrubiin is indicative of the comparative instability of the former compound to the more stable furanic form. Generally, under certain conditions the chemical treatment of 9,13–15,16-diepoxylabdane derivatives can converted them into the more stable furanic form, and no work has been done to study the effect of other substitution patterns in the C_11,12_ and C_14-17_ systems, however, many prefuranic structures have been isolated without their corresponding furanic forms [44,45,46,47]. The relation between premarrubiin and marrubiin needs to be investigated in the plant tissues using one of the available advanced techniques like high field solid-state NMR.

## 5. Chemical Aspects

### 5.1. Synthesis

Marrubiin was isolated in pure form in 1932 by Mercier and Mercier in a yield of 0.4%. The chemistry of marrubiin commenced in the last century and the molecular formula was established by Gordin, and its synthesis completed in 1970.

**Figure 1:**
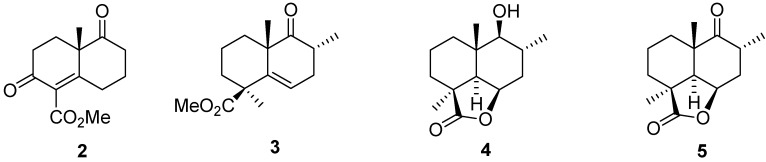
Synthesis intermediates of marrubiin (compounds **2–5**).

**Figure 2:**
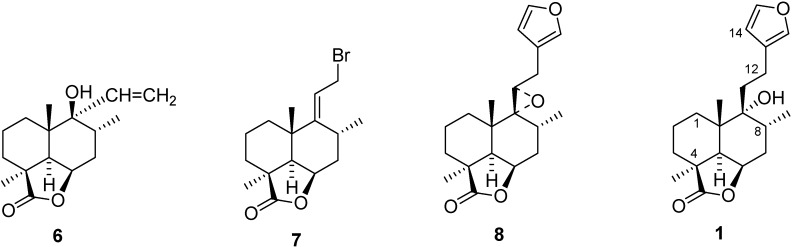
Synthesis intermediates of marrubiin (compounds **1**, **6–8**).

The synthesis of marrubiin (**1**) was achieved starting from the keto lactone **5** (Figure 1) which was prepared stereoselectively from the known keto ester **2** *via* **3** and **4** (Figure 1). Compound **5** on reaction with Li acetylide followed by reduction gave **6**, which gave **7** on treatment with PBr_3_ in pyridine (Figure 2). The bromide **7** was converted into the furanoepoxide **8** (Figure 2) by a reaction with 3-furanyl lithium followed by epoxidation. The final step, *i.e.*, the conversion of **8** into marrubiin (**1**) was achieved by reduction with lithium in ethylamine [48]. Its stereochemistry had been established by chemical transformations and confirmed by X-ray crystallography in 1982 [6,49].

### 5.2. Structure Modification

**Figure 3:**
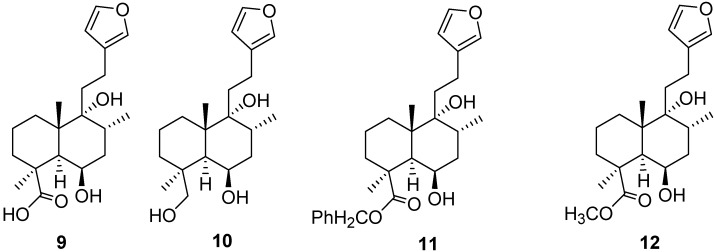
Different derivatives of marrubiin (compounds **9–12**).

There have not been many extensive structure-activity relationship studies made on derivatives of marrubiin despite its abundance and high pharmacological activity. Opening of the lactone ring using refluxing potassium hydroxide solution afforded the active marrubiinic acid (**9**, Figure 3) in a yield of ~70%. Pharmacological studies have shown that marrubiinic acid possesses antinociceptive activity against the writhing test, with an LD_50_ value of 12 µM/kg, being about 11-fold more active than the standard drugs (aspirin, paracetamol and morphine) [10]. Marrubenol (**10**, Figure 3) was obtained from marrubiinic acid in a yield of 98% after reduction with lithium aluminium hydride. Marrubenol and marrubiin were both found to elicit contractions evoked by high-KCl solutions in aortic segments thereby showing promise as a vasorelaxant. It was found that blocking the free acidic group of marrubiinic acid like in the esterified products **11** and **12** (Figure 3) reduced the biological activities, which indicates the major role of the free carboxylic group in contributing to the observed anti-inflammatory activities.

**Figure 4:**
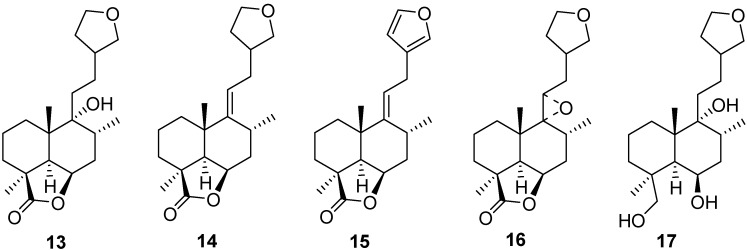
Different derivatives of marrubiin (compounds **13–17**).

On the other hand, during the course of structural elucidations performed in earliest studies, many derivatives have been prepared, e.g., compounds **13–17** (Figure 4) without any tests being conducted to determine their biological activities [50].

## 6. Pharmacological Aspects of Marrubiin

### 6.1. Toxicity Studies

According to the NIH PubMed website [50] marrubiin doesn’t demonstrate any cytotoxicity against 66 cancer cell lines, however on the other hand; *in vivo* experimental studies have documented a LD_50_ of 370 mg/kg body weight, for marrubiin [51]. Recent studies have showed a safety limit up to 100 mg/kg body weight when injected into mice [9].

### 6.2. Antinociceptive Activity

De Jesus *et al*. have reported on the antinociceptive effects of marrubiin and these were found to be dose-related. The antinociceptive properties were observed using both the systemic and oral routes and its action has sustained over a long period. The high potencies observed in the writhing test and formalin induced pain test suggest that marrubiin is acting by some peripheral mechanism. The antinociception induced by marrubiin was not reversed by naloxone when analyzed in the writhing test. In the hot-plate test, marrubiin did not increase the latency period of pain induced by the thermal stimuli [12].

### 6.3. Cardioprotective Activity

Although beneficial for cardiomyocyte salvage and to limit myocardial damage and cardiac dysfunction, the restoration of blood flow after prolonged ischemia exacerbates myocardial injuries. Several deleterious processes that contribute to cardiomyocyte death have been proposed, including massive release of reactive oxygen species, calcium overload and hypercontracture development or leukocyte infiltration within the damaged myocardium. Chemokines are known to enhance leukocyte diapedesis at the inflammatory sites. The diterpenoid marrubiin from *Leonotis leonurus* extracts was found to dampen the hypercoagulable and inflammatory state associated with obesity, therefore providing a cardioprotective role. It is clear that marrubiin has been reported to manifest its effects as an anti-inflammatory agent through the suppression of NF-κB signalling pathway. Chemokines recruit leukocytes to inflammatory sites; an agent that inhibits RANTES possesses cardioprotective action through its anti-inflammatory property [11]. The marrubiin extract from *L*. *leonurus* inhibited the secretion of RANTES, therefore marrubiin has the potential of being a cardioprotective agent as it inhibited platelet aggregation, hypercoagulation and the inflammation *in vivo*. This provided the first evidence that inhibition of CCL5/RANTES exerts cardioprotective effects during early myocardial reperfusion, through its anti-inflammatory properties. It was found to dampen the hypercoagulable and the inflammatory state associated with obesity, thereby providing a cardioprotective role.

### 6.4. Gastroprotective (Anti Ulcer) Activity

The treatment of gastric ulcers includes antacids and antisecretory drugs, mainly antagonists of histamine-2 receptors and proton pump inhibitors, which block the secretion of gastric acid. These treatments are effective, but may have many adverse effects, such as hypersensitivity, arrhythmia, impotence, gynecomastia and hematopoietic disorders. The search for new antiulcer therapies that can combine efficacy and lower toxicity like *M. vulgare* a plant used for treating various diseases, including gastric ulcers [10] becomes justifiable and warrants examination. The antiulcer activity of the methanolic extract obtained from leaves of *M. vulgare* and its main compound, the diterpene marrubiin, was examined by Paula de Oliveira A. *et al*. (2011). The gastroprotective activity of marrubiin was established through *in vivo* analysis on assays using different protocols (ulcers induced by ethanol/HCl and indomethacin/bethanecol) in mice [7]. In both models, marrubiin (25 mg/kg) produced a significant reduction when compared with the control group (*p* < 0.01). Marrubiin was found to contribute to an increase in the defensive mechanisms of the stomach through the production of prostaglandin synthesis [10] and the stimulation of the bronchial mucosa [6]. It is reported to have antiarrhythmic properties and may induce the cardiac irregularities. The results also demonstrated that the gastroprotection by marrubiin is related to the activity of NO and endogenous sulfhydryls, which are important gastroprotective factors that have vasodilator effects, thereby inhibiting gastric acid secretion. Pre-treatment with L-NAME revealed that the gastroprotective effect of marrubiin is strongly related to NO synthesis, an important endogenous transmitter released by the endothelial cells when mucosa is exposed to damaging agents.

### 6.5. Anti-Diabetic Activity

*Leonotis leonurus* an indigenous South African plant has been reported to be traditionally used to cure hypertension [12]. Jao evaluated the aqueous extract for its cardiovascular and hypotensive effects in rats and observed that the arterial blood pressures and heart rates of normal, anaesthetized spontaneously hypertensive rats were significantly reduced [52]. It was confirmed that the ethanolic extract of *L. leonurus* reduced plasma protein uptake and that marrubiin is responsible for the stimulation of insulin secretion in 1NS-1 cells of obese rat model. The anti-diabetic activity of marrubiin was studied *in vivo* using an obese rat model [4], which resulted in an increase in respiratory rate and mitochondrial membrane potential under hyperglycemic conditions. Marrubiin was found to increase insulin secretion and LDL-cholesterol in this study. In vitro analysis carried out on marrubiin confirmed the stimulatory index of INS-1 cells cultured under hyperglycemic conditions, and this was significantly increased in cells exposed to them. Furthermore the insulin and glucose transporter-2 gene expressions were significantly increased by marrubiin [9]. INS-1 cells cultured under hyperglycaemic conditions increased chronic insulin secretion by 1.5-fold relative to the normoglycaemic control (NGC) cells (*p* < 0.05).

### 6.6. Antispasmodic and Ca^2+^ Antagonist Potential

Hussain *et al*. have demonstrated the antispasmodic potential of marrubiin when administered to rabbit jejunum. It caused concentration-dependent relaxation and high K (80 mM)-induced contractions, very similar to that caused by verapamil, indicating that marrubiin exhibits spasmolytic activity possibly mediated through the Ca^2+^ channel blocking action [6].

### 6.7. Antioedematogenic Activity

Marrubiin was evaluated against carregeenan-induced oedema because it is known to be involved in the release of different inflammatory agents. Its test is highly sensitive to non-steroidal anti-inflammation drugs, and it has long been accepted as a useful phlogistic tool for investigating new anti-inflammatory drugs. Marrubiin has significant inhibitory effects on ear oedema. It has been established that kinins are pro-inflammatory peptides that mediate a variety of pathophysiological responses. These actions occur through the stimulation of two pharmacologically distinct receptor subtypes viz; B1 and B2. The fact that there is strong kinin participation in oedematogenic components of the formalin test indicated that marrubiin reinforces the hypothesis of the role of kinins in the marrubiin anti-inflammatory actions. However, the inhibitory activity of marrubiin on pro-allergic agents such as serotonin and histamine was tested with the compound 4880, which has significant inhibitory effects on compound 4880-induced ear oedema, indicating the possible existence of stabilizing mastocyte membranes, analogous to an allergic status. Marrubiin showed a dose-dependent effect when the oedema was induced by these agents, suggesting non-specific receptor actions, following the release of serotonin and histamine. Various pro-inflammatory agonists were tested against marrubiin in oedema development using microvascular extravasation of Evans blue dye. The results demonstrated that marrubiin presents global inhibitory effects on different phlogistic agents, including histamine, and to a lesser extent, substance P, participating to a greater or lesser degree in the inflammatory process. Antioedematogenic activity of marrubiin was analyzed in a model of microvascular leakage in mice ears. The results as reported by Stulzer show that it exhibits significant and dose-related antioedematogenic effects. The other phlogistic agonists, such as prostaglandin E2 (PGE2), caused inhibition of less than 50%. In addition, marrubiin (100 mg/kg) significantly inhibited the OVO-induced allergic oedema in actively sensitized animals (maximal inhibition 67.6 ± 4%) [2,9].

### 6.8. Analgesic Activity

Success was obtained in reducing the lactone ring of marrubiin which led to the formation of marrubiinic acid (**9**) and two esterified derivatives exhibited significant analgesic effect using the writhing test in mice. Marrubiinic acid showed higher activity and excellent yield, and its analgesic effect was confirmed in other experimental models of pain, suggesting its possible use as a new and useful analgesic agent [10].

### 6.9. Anticoagulant and Antiplatelet Activities

Anticoagulant and antiplatelet activities of marrubiin have also been detected. According to Mnonopi *et al.* marrubiin was found to significantly prolong activated partial thromboplastin time (APTT) with fibrin and D-dimer formation being drastically decreased TNF-α and RANTES secretion were also reduced by the extract and marrubiin when measured in the obese rat model relative to the controls. Calcium mobilization and TXB2 synthesis were also suppressed [4]. Several diterpenoids from *M. cylleneum* and *M. velutinum* were tested for their cytotoxic effects against various cancer cell lines and their immunomodulating potential in human peripheral blood mononuclear cells were standardized. The results showed a differential cytotoxicity of compounds as well as their ability to improve selected lymphocyte functions [22].

### 6.10. Vasorelaxant Potential

The result of Khan *et al.* revealed that marrubiin present among other constituents from *Phlomis bracteosa* exhibited vasodilator action *via* a combination of endothelium-independent Ca^2+^ antagonism and endothelium dependent NÃ¹-nitro-L-arginine methyl ester-sensitive nitric oxide-modulating mechanisms [53]. The crude extracts of the aerial parts of *M. vulgare* showed appreciable potently in vitro inhibition of KCl-induced contraction of rat aorta [11] thereby indicating vasorelaxant properties in this medicinal plant extract.

## 7. Conclusions

Marrubiin exists in high concentrations in many traditionally important Lamiaceae species and has demonstrated excellent pharmacological properties with high safety margins in different inflammation models. The low turnover, high stability and little catabolism further indicate its importance and it comparatively, little has been done concerning its chemical modifications and the examination of the associated bioassay data merits continued chemical investigation.
